# Supplementary material for: AMSD: The Australian Message Stick Database
Source: PLoS One. 2024 Apr 10;19(4):e0299712. doi: 10.1371/journal.pone.0299712 (PMC11006167; doi:10.1371/journal.pone.0299712)
Supplement: Attachment — Submitted filename: 20240209-Response to reviewers.pdf [file pone.0299712.s001.pdf]

09.02.2024

Dear Editor,

Our manuscript, “AMSD: The Australian Message Stick Database”, is hereby resubmitted with revisions. We thank the reviewers for their comments.

Here is the revised financial disclosure, as you requested: “The lead author (Piers Kelly) receives salary and project funding specifically for the research described in this paper. He is funded by an ARC Discovery Early Career Researcher Award with the grant number DE220100795. No other author has received specific funding for this work. The funder had no role in study design, data collection and analysis, decision to publish, or preparation of the manuscript”.

We have accepted all the reviewers comments and addressed them in line with their suggestions. A table listing all recommended changes is appended below. In only two cases (Reviewer 2, comments 16 and 20 below) do we believe the reviewer made structural suggestions based on a misreading of the existing subheading structure. These have not been implemented. Comment 33, asking for more detail about the second phase of research under the direction of co-author Lorina Barker was rejected by her and her First Nations associates. Their project is in its early stages and does not exclusively involve message sticks.

We have made a small number of minor corrections outside the reviewers’ comments. These are: typographical errors, spacing errors and the replacement of images with higher resolution versions that became available during the review period. We also changed “engraved mobile objects” on p6 to “engraved Upper Palaeolithic objects” so that readers understand that these are archaeological artefacts of a specific period and not generic mobile objects. All other changes are in direct response to the reviewers’ comments.

We are all happy for peer review history to be published, keeping in mind, however that there is Indigenous co-authorship and that the decision will need to be made in accordance with the PLoS policy on vulnerable populations.

Yours Sincerely,

Piers Kelly

	Revision	Requested by	Action	Status	Description of change
1	STYLE: Please ensure that your manuscript meets PLOS ONE's style requirements, including those for file naming. The PLOS ONE style templates can be found at: https://journals.plos.org/plosone/s/file?id=wjVg/PLOSOne_formatting_sample_main_body.pdf and https://journals.plos.org/plosone/s/file?id=ba62/PLOSOne_formatting_sample_title_authors_affiliations.pdf	Editor	Updated manuscript style including file names	Done	PK has gone through the manuscript and checked off all style and formatting requirements. In addition he has regularised the Microsoft Word formatting styles to make navigation easier. Files have been renamed. We have also found higher resolution versions of Figs 6, 8 and 10.
2	STYLE: Thank you for stating the following financial disclosure: “The lead author (Piers Kelly) receives salary and project funding specifically for the research described in this paper. He is funded by an ARC Discovery Early Career Researcher Award with the grant number DE220100795. No other author has received specific funding for this work.” Please state what role the funders took in the study. If the funders had no role, please state: "The funders had no role in study design, data collection and analysis, decision to publish, or preparation of the manuscript." If this statement is not correct you must amend it as needed. Please include this amended Role of Funder statement in your cover letter; we will change the online submission form on your behalf.	Editor	Updated disclosure	Done	This is correct and has been added to the cover letter
3	We note that Figure 11 in your submission contain [map/satellite] images which may be copyrighted. All PLOS content is published under the Creative Commons Attribution License (CC BY 4.0), which means that the manuscript, images, and Supporting Information files will be freely available online, and any third party is permitted to access, download, copy, distribute, and use these	Editor	Updated CC-BY information in manuscript	Done	This image is from the AMSD so the data is free to reuse with attribution. The underlying raster layer is from OpenStreetMap and is released under CC-BY-SA. I have re-extracted the image in order to include the CC-BY information and updated the caption as follows: "Fig 11. Sites of origin of message sticks coded according to descriptive adequacy. Map extracted from https://amsd.clld.org/ on 16 January

	materials in any way, even commercially, with proper attribution. For these reasons, we cannot publish previously copyrighted maps or satellite images created using proprietary data, such as Google software (Google Maps, Street View, and Earth). For more information, see our copyright guidelines: http://journals.plos.org/plosone/s/licenses-and-copyright.				2024. All data and layers are released under CC-BY-SA."
4	REFERENCES: Please review your reference list to ensure that it is complete and correct. If you have cited papers that have been retracted, please include the rationale for doing so in the manuscript text, or remove these references and replace them with relevant current references. Any changes to the reference list should be mentioned in the rebuttal letter that accompanies your revised manuscript. If you need to cite a retracted article, indicate the article's retracted status in the References list and also include a citation and full reference for the retraction notice.	Editor	Checked references	Done	No references need to be revised
5	CONTENT: Some statements and generalizations in relation to museums are problematic, such as stating the Australian museums have withdrawn collections from the public; eg. Line 433 begins discussion of current protocols in museums but fails to mention these are imposed/informed practice from the document commissioned by the Australian Museums and Galleries Association from Indigenous lawyer Terri Janke - FIRST PEOPLES: A ROADMAP FOR ENHANCING INDIGENOUS ENGAGEMENT IN MUSEUMS AND GALLERIES - which emerged as a consequence of lobbying by Indigenous arts practitioners in the GLAM sector. While the	Reviewer 1	Addressed as requested by reviewer	Done	This clarification has been added in full to the relevant section (lines 478 to 502)

	intention is to give greater say to Indigenous people, it has at the same time created new issues in terms of access without permission of TOs, which is well explained in the discussion - you don't know who to ask if you don't know where it comes from. However the discussion in the manuscript sounds like museums have just decided in 2019 to close down access - more context or explanation is needed.				
6	TERMINOLOGY: Terminology in places is cumbersome such as the use of the term 'conserved' in relation to objects in museums. That is one of many responsibilities of museums in relation to objects so suggest using 'held by museums' or a similar term.	Reviewer 1	Addressed as requested by reviewer	Done	All instances of 'conserve' or 'conserved' are now replaced with more appropriate terms. Eg, 'held', 'curated', or 'stored'.
7	TERMINOLOGY: The other term is 'filed' - eg. in Line 50 the authors state the message sticks were 'filed' by an 'Indigenous recipient' - unsure that best describes that fact they are tied together. And do we know the "Indigenous recipient' actually tied these together?	Reviewer 1	Addressed as requested by reviewer	Done	Updated to "Fig 1. A set of five message sticks linked with string, held at the Grassi Museum Leipzig "
8	TERMINOLOGY: Similarly, in Line 235, the authors discuss 'thicker descriptions' - does this mean more detailed descriptions?	Reviewer 1	Addressed as requested by reviewer	Done	Replaced with "more detailed" (N.B. 'thick' is a well established term in anthropology to describe descriptions that have greater depth as opposed to simply more detail)
9	CONTENT: Line 266 raises the issue of 'centres of diffusion' which is not addressed anywhere in the manuscript so maybe is best left to explore in more detail in a paper on the actual message sticks	Reviewer 1	Addressed as requested by reviewer	Done	Deleted the sentence: "This could permit historical linguists to trace the spread of message sticks across historical time and perhaps isolate centres of diffusion"
10	CONTENT: in Line 278 the absence of 'overtly racist' language in museum records is a puzzling - why mention it if it is absent - again needs more context and beyond the scope of this paper?	Reviewer 1	Addressed as requested by reviewer	Done	Deleted: "Though individual museum records of message sticks may not appear to be overtly racist in character in terms of, for example, archaic and insulting terminology, " and retained remaining portion of the sentence: "Indigenous perspectives are

					rarely present in any of the metadata fields"
11	CONTENT: I question the use in Line 38 of 'oblong' to describe the shape of message sticks. The figure accompanying this shows cylindrical shaped message sticks - definitely not oblong! So are they oblong (essentially rectangular with uneven sides and flat surfaces) and/or cylindrical? Overall, it is important to be clear the authors are giving a general not comprehensive overview of the form and its use as this paper is focused on the database	Reviewer 1	Addressed as requested by reviewer	Done	Deleted: "oblong-shaped" and added "generally". Final sentence: "Message sticks are hand-made wooden objects that are generally either flattened or cylindrical..."
12	CONTENT: A minor point is in Line 68 which states that message sticks are 'prominently displayed' yet it is said they can be 'in a net bag' which would mean they would not be seen in that instance.	Reviewer 1	Addressed as requested by reviewer	Done	Deleted: "in a net bag,"
13	CONTENT: I believe the article meets the publication criteria of PLOS ONE. However, I have some comments more focused on the interpretation of the effects of this database, presented as suggestions for future works rather than critiques of the manuscript. For instance, given that message sticks were in proper use in Arnhem Land until the 1970s, is there anyone among the TOs up there who still possesses traditional knowledge? It would be valuable to include them in the study.	Reviewer 2	Addressed as requested by reviewer	Done	Note that these contemporary knowledge holders are included in the study. Updated sentence in the abstract: "It contains images and data for over 1500 individual message sticks sourced from museums, and supplemented with information derived from published and published manuscripts, private collections, and from field recordings involving contemporary Indigenous consultants." Later, under the subheading Country-centred design we have added: "These higher-value records include relatively recent fieldwork on message sticks involving living Knowledge Holders in the Top End, as well as historical documentation carried out under the direction of Indigenous messengers who are now deceased."

14	CONTENT: the issue of missing information is common and distressing in museums. How do the authors plan to address this beyond interactive comments? Additionally, is there a plan for content moderation that allows input from everyone regarding the items listed in the database?	Reviewer 2	Addressed	Done	Added to the end of section 'Purposes of the database', the following sentence: "While these issues cannot be comprehensively addressed by the AMSD itself, users can nonetheless provide commentary, contribute new information or report errors through the ‘Give feedback’ bell icon at the top of each entry (see Fig 4). "
15	CONTENT: p.2, l.25: typo. Do you mean ‘published and unpublished manuscripts’?	Reviewer 2	Addressed as requesed by reviewer	Done	Fixed, thank you that was a glaring typo
16	STRUCTURE: p.3, l.41: It would be helpful to first mention the source of the sample before providing details about the objects. This includes specifying the museum(s), the quantity of objects from each museum, and whether the measurements are from the museum's archive or were taken by the authors. I notice this information is already available below, so I recommend moving these sections here for better organisation.	Reviewer 2	Considered and rejected	Not done	Not sure we fully understand this remark. The sentence in question immediately identifies the source. Further, we don't think that bringing in detailed information about each museum is appropriate in an Introduction which should focus on generalites. We believe this may have been a simple misreading so we have made no change.
17	REFERENCES: p.3, l.38-onwards: it might be useful to add more literature references to this section.	Reviewer 2	Addressed as requested by reviewer	Done	Yes. Note that there are hundreds of supporting references for this information so we have referenced a single review paper that summarises the most significant.
18	CONTENT: p.5, l.74-74: ‘After use, message sticks have been known to be repurposed as other objects’. Can you elaborate on which were these other uses? Just a couple of examples to give an idea of the meaning the message sticks had during as well as after their use.	Reviewer 2	Addressed as requested by reviewer	Done	Changed to: "After use, message sticks have been known to be destroyed, filed in sets (see Fig 1), or repurposed as other objects such as hairpins [6] or clapsticks (AMSD IDs: SAM_A_52915, SAM_A_52916)."
19	CONTENT: p.5, l.76: I question the use of the word ‘settlers’ (also above in l.64). Apart for being not a very appreciated word, it is in my opinion too vague. Are them anthropologist, photographers, priests, famers..?	Reviewer 2	Address as requested	Done	We acknowledge the controversy over this word and have found synonyms for the instances identified by the reviewer. We have retained 'settlers' in the abstract since it is the most inclusive lexical choice in this instance.

20	STRUCTURE: p.11, l.208-onwards: I believe the whole sections ‘Message sticks from collecting institutions’ and ‘Message sticks described in documentary sources’ should appear much earlier in the manuscript (I would suggest just after the historical accounts on message sticks).	Reviewer 2	Considered and rejected	Not done	These two sections—‘Message sticks from collecting institutions’ and ‘Message sticks described in documentary sources’—are actually subsections of the section 'Data sources'. In other words, the sections are *not* about message sticks generally but merely those that are a) in the AMSD, and b) are derived from these two distinct source types. Rather than providing introductory commentary on message sticks, they are in this section of the manuscript so that users of the database can understand the data itself. We believe that when the manuscript is properly typeset it will be easier to see how these are subsections of 'Data sources'.
21	CONTENT: p.12, l.217-223: Misidentifying artifacts in museums is a widespread issue. Is the creation of the database, as a project rather than just the database itself, aimed at addressing this problem? Or is it primarily a means of ensuring that the database exclusively includes message sticks? It would be beneficial if the authors shared their thoughts on this matter. Additionally, what about objects that are indeed message sticks but are identified differently in museum archives? Does the project associated with the database intend to tackle this concern too?	Reviewer 2	Addressed as requested by reviewer	Done	We have rewritten the last sentences of this section thus: "In some cases, hairpins, tjurungas (see ‘Cultural sensitivity’ below) and other morphologically similar objects are misidentified as ‘message sticks’ and several of these have been found and corrected by the project team. However, it is a shortcoming of the database that other underdocumented objects—labelled in museum or documentary sources as ‘message sticks’ but with limited contextual information— may prove to be false positives if and when more reliable sources come to light. Harder to detect are false negatives: objects in institutions that are message sticks but are mislabelled as other kinds of artefacts. Every effort is made to ensure that the objects are true message sticks before they are included but this cannot be guaranteed. One way that we have attempted to compensate for such limitations within the database is by coding entries according to descriptive adequacy so that users can filter out less reliable entries (see ‘Country-centred design' below). "

22	CONTENT: p.13, l.238-240: 'The benefit of triangulating sources in this way is that seven 'orphaned' museum objects of unknown meaning or provenance have already been identified by the project on the basis of non-museum sketches and descriptions'. This is very remarkable. Could the authors provide additional insights into the process of triangulation? How did they determine that the museum objects left orphaned were the same as those described in the accounts? While it might be evident when comparing drawings alongside objects, the identification process seems less straightforward when initiated solely from written descriptions.	Reviewer 2	Done	Done	This was unclear since we have never used written descriptions in isolation but rather in conjunction with sketches. The relevant sentence is updated: "The benefit of triangulating sources in this way is that seven 'orphaned' museum objects of unknown meaning or provenance have already been identified by the project on the basis of non-museum sketches, in conjunction with written descriptions (see Figs 7–10 for examples). "
23	CONTENT: p.22, l.436-438: I recommend including a brief reflection on the impact of having "impossible standards." Without these standards, in-person visits to the objects could be facilitated, allowing for the capture of new, high-quality pictures. This would prove invaluable for communities to examine 'orphan' items and determine if they rightfully belong to their country. At times, museum photos may not suffice for this purpose, even if the database theoretically makes them accessible to the Traditional Owners.	Reviewer 2	Addressed as requested by reviewer	Done	Added this sentence: "The limitations on in-person visits also reduce opportunities for communities to examine or photograph unprovenanced (or underprovenanced) objects with the aim of determining whether or not they rightfully belong to their Country. "
24	DATA: - There appears to be some inconsistency between the number of artefacts in the database as described in the Abstract (1490), and on the website landing page (1413; https://amsd.clld.org/)	Reviewer 3	1. Synced with CLLD; 2. updated all the stats/numbers	Done	This was due to the fact that data entry interface (containing the complete data) can only be periodically synced with the public interface so the numbers don't always correspond. Note also that, since submission, our team has added a small number of new entries to the dataset. We have therefore decided a) to resync the data so that the public interface matches the data-entry interface, and b) update the relevant figures in the paper and any calculations derived from them. (Please note that

					none of these corrections have a bearing on arguments or analysis made in the paper, since the paper is descriptive and has no hypothesis.)
25	CONTENT: - Introduction: for avoidance of doubt, it would be worth stating up front the definition of 'message sticks' in the database, and how they differ from secret or sacred objects with restricted access (eg tjurunga)	Reviewer 3	Addressed	Done	This is addressed in comment 21 above but we have also revised an introductory sentence thus: "What makes a message stick a message stick is the fact that it is a central material token in a public long-distance communicative event, a fact which distinguishes it from restricted objects. "
26	CONTENT: Introduction: in a similar vein, can the authors also clarify (up front) the role of Aboriginal and Torres Strait Islander communities in this study, and whether any ethics processes have been followed (if required)?	Reviewer 3	Addressed as requested by reviewer	Done	Author Lorina Barker is a Muruwari and Wangkumara woman with cultural expertise, as well as a university scholar. In the introduction we have now included "compiled by Indigenous and non-Indigenous scholars" and updated another sentence to read: "Muruwari and Wangkumara scholar Lorina Barker, a contributor to this paper, explains that uninterpreted message sticks are still undergoing their first journey and will find their way home only when they are materially reunited with their Country". The Acknowledgments outline sall the Indigenous expertise on the project. We have added to the acknowledgments: " Ethics approval for the project was granted by The University of New England."

27	CONTENT: - Introduction: it would be worth noting that message sticks are part of a long and varied tradition of engraved and marked wooden items across the continent, including Aboriginal culturally modified trees (eg Spry et al 2023 Investigating Wiradjuri marara (carved trees or dendroglyphs) and dhabuganha (burials), Australian Archaeology journal, amongst other articles)	Reviewer 3	Addressed as requested by reviewer	Done	Dendroglyphs are probably not the best example since they are not mobile and are specific to one region, but we have added this sentence to the Introduction: " However, it is clear that provenanced objects are found within diverse semiotic contexts that have their own recognisable stylistic conventions expressed, for example, through rock art, body paint, sand-drawings, shield designs, or tree carving."
28	CONTENT: - Introduction: it could be made a clearer in this section if the authors are arguing that the markings on message sticks have any symbolic meaning or not	Reviewer 3	Addressed as requested by reviewer	Done	We have added the word 'meaningful' to this sentence: "Designed as communication aids, they are marked with meaningful motifs that are engraved or painted, or a combination of both" but note that we have more explicitly made this point already in this sentence "Markings on the wood reinforce the legitimacy of the communication and typically correspond to individual elements of an accompanying spoken message (Fig 2). "
29	CONTENT: - Lines 115-116: "…subject to the control and approval of community representatives". Do you mean Aboriginal and Torres Strait Islander community representatives? Please clarify if so. It would also be interesting to know more about how this is envisaged to work	Reviewer 3	Addressed as requested by reviewer	Done	We have added the crossreference: "approval of Indigenous community representatives (see 'Access issues and Indigenous Cultural and Intellectual Property' below); " and we have expanded the end of that section as follows: "One response to the present situation has been the development of the Mura Maarni framework by the Taragara Research Group, a research partner of the AMSD [41]. The model emphasises the slow development of community relationships and reciprocal obligations with collecting institutions, and stresses the inclusion of cultural metadata with curated objects, respect for data sovereignty, and the minimisation of administrative barriers for community access. It is this devolved, open and adaptive framework that continues to inform our interactions between community representative organisations and the

					database. Since each community brings its own concerns, values and interests to the data, there is no one-size-fits-all policy when it comes to representing cultural information. "
30	CONTENT: - Line 283: it would be worth noting that a historical focus on dimensions, techniques and motif styles, and an absence of Aboriginal people, perspectives and voices, is characteristic of most previous studies of Aboriginal cultural heritage in Australia (eg rock art, Aboriginal culturally modified trees (Etherbridge 1918; Lindsay Black 1941))	Reviewer 3	Addressed as requested by reviewer	Done	Added this sentence: "While this state of affairs is commonplace in colonial descriptions of Indigenous material and symbolic culture, these realities have presented a dilemma in the compilation of the AMSD. "
31	CONTENT: - Figure 11: requires a legend	Reviewer 3	Addressed per comment 3 above	Done	See response to comment 3

32	CONTENT: - Line 369-380: can the authors include references to support these statements? Is this view also shared across Aboriginal and Torres Strait Islander communities?	Reviewer 3	Updated to include references for each statement in text and clarifying statement about variation	Done	Thirteen references added to this section (but note that the seven existing references to entries in the AMSD also support these claims). Added: "The two most general functions of message sticks—as visual tokens of a messenger's permission to travel, and as the authentication of a publicly witnessed communication— are known in all regions of Australia where message sticks are reliably documented in close consultation Aboriginal knowledge holders, despite differences in the ways such interactions may unfold in practice."
33	CONTENT: - Line 476-478: can more detail be provided here about this second phase of research? This description currently appears a little vague	Reviewer 3	No change	Not done	Lorina Barker and Taragara Research reviewed this passage but did not see the benefit of describing the Songline of Country project in detail since it is not specific to message sticks. I (Piers Kelly) agree with this position.
34	CONTENT: - Acknowledgments: the authors may wish to include acknowledgment of all Aboriginal and Torres Strait Islander peoples who created and used message sticks, including those included in the database	Reviewer 3	Addressed as requested by reviewer	Done	We have added this sentence to the Acknowledgments: "We acknowledge the individual creators of all the message sticks included in the Australian Message Stick Database. In almost all cases their names are not recorded. Named creators in the database are as follows with recording spellings preserved, with the Country or clan associated with their message sticks is included in parentheses: ". Following this we have listed 56 names of named creators.

35	CONTENT: General comment: it would be helpful for the reader to know if this project has, or will, include Aboriginal and Torres Strait Islander community consultation - particularly for well provenanced examples in the database? Noting what the authors state in the section 'Country-centred design'). Can the authors reflect more clearly on how steps could be taken to ensure this database is used in the future for culturally appropriate research?	Reviewer 3	Addressed as requested by reviewer	Done	We wish to point out that the database already includes clear guidance on the front page about culturally appropriate use, in line with best practice as it relates to Australian cultural materials. However, we have added the following statement: "We note that ethical considerations that pertain to Indigenous cultural knowledge in Australia can change quickly in response to evolving social and political conditions. Researchers using this resource are advised to consult the latest guidance from AIATSIS and local Indigenous communities. Our policies on community consultation and interactions with collecting institutions are set out at https://messagesticks.com.au/. "
36	CONTENT: - General comment: can the authors include discussion of how his study considers FAIR and CARE principles in terms of Indigenous data (https://ardc.edu.au/resource/the-care-principles/)?	Reviewer 3	Addressed as requested by reviewer	Done	Added this sentence: "Recently, scientists and Indigenous cultural workers have looked for ways to combine the FAIR (Findable Accessible Interoperable Resuable) and CARE (Collective benefit, Authority to Control, Responsibility, Ethics) principles with the aim of promoting a more ethically sustainable and scientifically rigorous management of datasets [49]. While the work is ongoing, the AMSD has sought to adhere to these general principles by making its (non-restricted) data available in a standardised way and identifying Indigenous data owners in the entries, wherever possible. A related response has been the development of the Mura Maarni framework ...."
37	CONTENT: - General comment: as this is an international journal, can the author provide more detail on comparable items from other countries? Noting that some comparison is already included	Reviewer 3	Addressed as requested by reviewer	Done	We have included every possible comparator to message sticks that is known to us except the use of mou khé by the Hmong which is now included.

38	CONTENT: I was unable to view the entries through either of the two links that accompany this manuscript. I'm unsure if this is because I reviewed the article on a non-PC device with iOS software. The second link asked me for login information, which I do not possess. I encourage the authors to look into this to ensure everyone can view and access all content and website functions through both links. It is for this reason I have stated that data have not been made available for this study	Reviewer 3		Done	The complete list of links in the paper is: https://amsd.clld.org/; https://amsd.clld.org/contributions/BM_Oc1901_1221_20#4/-22.28/144.58; https://glottolog.org/; https://github.com/khipulab/open-khipu-repository; https://messagesticks.com.au/; http://ehrafworldcultures.yale.edu/ehrafe/; https://cdhr.cass.anu.edu.au/research/projects/occams . We have tested all of these on OS and iOS devices with a variety of browsers and found no issues.
39	GENERAL: PLOS authors have the option to publish the peer review history of their article (what does this mean?). If published, this will include your full peer review and any attached files.	Editor		Not done	We are happy for peer review to be published but leave it up to PLoS editors to decide, bearing in mind that there is Indigenous content and co-authorship
